# Current News

**Published:** 1900-05

**Authors:** 


*Secretary.*

---

### Current News.

### NATIONAL DENTAL ASSOCIATION.

The meeting of the Association which was to have been held June 26, at Old Point Comfort, has been changed by vote of the members to Tuesday, July 10. Reports from officers of sections indicate that a very high order of work will be done at this meeting. Men have been selected to write papers because of their peculiar fitness for the undertaking, and enough of those invited have accepted to insure a full programme. A special feature will be clinics, under the supervision of Section III. It is hoped that the attendance will be as large as it was last year at Niagara.

B. Holly Smith,

April 9, 1900. *President.*

---

### AMERICAN MEDICAL ASSOCIATION, SECTION ON STOMATOLOGY.

The next meeting of the American Medical Association will be held at Atlantic City, June 5 to 8, 1900. The Section on Stomatology presents the following programme:

### SYMPOSIUM ON DENTAL EDUCATION.

Relations of Dental and Oral Surgery to General Medicine: Professional Status of Properly Educated Practitioners of Dental and Oral Surgery, Dr. N. S. Davis, Sr.; Preliminary Qualifications, Dr. J. Taft; Course of Study, Dr. W. A. Evans; Methods of Teaching (Didactic or Recitational), Dr. A. H. Peck; Shall the Dental Student be educated independently of General Medicine? Dr. G. V. I. Brown; Is Medical Education a Necessary Qualification for Dental Practice? Drs. Alice Steeves and R. R. Andrews; The Practical Value of a Medical Education in Dental Practice, Dr. W. B. Hill; Technical Training *vs.* Theoretic, Dr. John S. Marshall; Should the Medical Undergraduate be instructed in the Principles of Dentistry? Dr. M. L. Rhein; Post-Graduate Study in Dentistry, and Degrees therefor, Dr. W. E. Walker; Handwriting upon the Wall: What does it portray? Dr. A. E. Baldwin; Limitations, Dr. Eugene S. Talbot.

### SYMPOSIUM ON INTERSTITIAL GINGIVITIS, OR SO-CALLED PYORRHŒA ALVEOLARIS.

Etiology, Dr. G. Lenox Curtis; Neurotic Affections, Dr. J. G. Kiernan; Indigestion Auto-intoxication, Dr. Eugene S. Talbot; Chemical Factors in Etiology, Dr. W. L. Baum; Constitutional Treatment, Dr. J. H. Salisbury; Local Treatment, Dr. M. H. Fletcher; So-called Glands in the Peridental Membrane, Dr. M. H. Fletcher; The Evolution of Decay continued, Dr. Arch C. Hart; Co-operation of the Public Schools in Teaching. Good Teeth,

Good Health. Whatever we wish to introduce into the life of a nation must be introduced into its schools, Dr. Richard Grady; Subject to be announced, Dr. V. A. Latham.

The Section on Stomatology will meet at Hotel Senate. The officers of the section invite all to be present and to take part in the discussions.

Those who wish to join the Association must obtain credentials from their State or local Dental Societies and pay five dollars to the Secretary of the Association. This will entitle them to the Journal for one year.

Accommodation can be had by writing F. B. Cook & Son, Hotel Senate.

EUGENE S. TALBOT,
*Secretary Section on Stomatology.*

---

### NATONAL ASSOCIATION OF DENTAL FACULTIES.

THE National Association of Dental Faculties will meet at Old Point Comfort, Va., on the afternoon of July 13, 1900.

J. H. KENNERLY,
*Secretary.*

---

### NEW YORK STATE DENTAL SOCIETY.

THE thirty-second annual meeting of the New York State Dental Society will be held in the New Centennial Hall, corner Lodge and Pine Streets, Albany, N. Y., May 9 and 10.

The following programme is announced: President's Address, F. Le Grand Ames, D.D.S.; Correspondent's Report, R. Ottolengui, M.D.S.; Report of Committee on Practice, H. D. Hatch, D.D.S.; Essays, F. A. Capron, D.D.S., Toronto; Joseph Head, D.D.S., Philadelphia; Edward C. Kirk, D.D.S., Philadelphia; S. B. Palmer, M.D.S., Syracuse; Chas. H. Barnes, D.D.S., Syracuse; Milton F. Smith, D.D.S., New York.

F. LE GRAND AMES, *President.*
W. A. WHITE, D.D.S., *Secretary.*

### NATIONAL ASSOCIATION OF DENTAL EXAMINERS.

In consequence of a contemplated new movement by the Association, with the probability of considerable benefit both to the State Boards and the more advanced colleges whose educational standards are high, the secretary most earnestly requests from the officers and members of the several State Boards in the United States and Territories a new list of officers and members.

An early compliance with this request will be most heartily appreciated.

Charles A. Meeker, D.D.S.,
*Secretary.*

29 Fulton Street, Newark, N. J.

---

### KENTUCKY STATE DENTAL ASSOCIATION.

The thirtieth annual meeting of the Kentucky State Dental Association will be held in Louisville, beginning May 29, 1900, at nine A.M., and continuing three days.

Following is the preliminary announcement of papers to be read:

Some Advantages of Non-Cohesive Gold, Dr. J. R. Clayton; Amalgam: Its Preparation, Instruments, etc., Dr. W. E. Harper; Oral Manifestations of Syphilis, Dr. T. C. Evans; X-Rays in Dentistry, Dr. L. E. Custer; Subject to be given, Dr. S. A. Donaldson; Malaria as a Cause of Secondary Hemorrhage in Extraction of Teeth, Dr. J. P. Shaw; Orthodontia, Dr. C. DeWitt Lukens; Orthodontia, Dr. E. D. Rose; One of the More Especial Duties of the State Dental Association, Dr. J. L. Sutphin; Care of Deciduous Teeth, Dr. J. F. Meadors; Subject to be given, Dr. I. B. Howell; Dental Education, Dr. Theo. Menges; Practical Dentistry, Dr. E. T. Barr; Troublesome Cases in Bridge-Work, Dr. U. D. Hulick; Teeth, Dr. W. S. Williams; Metallo-Plastic Work and backing Porcelain Teeth, Dr. R. C. Brophy; The Status of Mechanical Dentistry, Dr. O. G. Wilson; Disease of the Antrum, Dr. Adolph O. Pfingst; The Reproduction of Gum Tissue in the Inter-Proximam Space, Dr. Geo. T. Carpenter; Subject to be given, Dr. J. H. Baldwin; Subject to be given, Dr. A. H. Peck; The Importance of Proper Physical Diagnosis in the Practice of Dental Surgery, Dr. J. Y. Crawford; Gold Filling *vs.*

Gold Crowns, Dr. W. T. McLean; Antiseptics and Disinfectants, Dr. Geo. W. Cook; What Efforts are we using to better the Profession? Dr. M. H. Dailey; Cast Aluminum Dental Plates, Dr. Willard Streetman.

CLINICS.

Oral Surgery, Dr. Wm. H. G. Logan; A Method of backing up Porcelain Crowns, Dr. E. D. Rose; Removal of Dental Pulp Surgically, Dr. J. Y. Crawford; Soft Gold Filling, Dr. P. A. Pennington; Metallo-Plastic Work, Dr. R. C. Brophy; A Compound Gold Filling Crown and Posterior Proximal with Matrix, Dr. B. Oscar Doyle; Porcelain, Dr. H. J. Goslee; Immediate Nerve-Extraction with Eucaine by Pressure and Root-Filling, Dr. S. A. Donaldson; Orthodontia, Dr. C. DeWitt Lukens; De Trey's Gold, Dr. C. K. Runyon; Orthodontia and Exhibit, Dr. Frank L. Smith; Contour Fillings with Soft Gold on Models, Dr. G. S. Junkerman; The Use of Snow Face Bow in taking a Base Plate Bite, Dr. J. Q. Bryam; Subject to be given, Dr. J. R. Clayton; A Few Cases in Orthodontia, Dr. J. S. McClurdy; Soft Gold Filling, Dr. Henry Pirtle; Subject to be given, Dr. W. E. Grant; Combination Gold Filling, Dr. E. L. Sanders; Open-Faced Crowns, Dr. B. G. Reese; Extraction of Teeth Under Local Anæsthesia, Dr. F. R. Wilder.

The following gentlemen will give clinics, subjects to be given: Dr. G. C. Roberts, Dr. F. L. Klingman, Dr. W. W. Barnes, Dr. A. B. Weaver.

Twelve firms have secured space for displays.

The committee, in addition, have under arrangement other important clinics, and are making strenuous efforts to make this the best meeting ever held in the State and well worthy your attendance. Members of the profession are cordially invited.

F. I. Gardner, D.D.S.,
*Secretary.*

---

### DENTAL COMMISSIONERS OF CONNECTICUT.

The Dental Commissioners of Connecticut will meet at the Capitol in Hartford, Monday and Tuesday, May 14 and 15, 1900, for the examination of candidates for license and attend to any business proper to come before them.

Practical examination in operative and prosthetic dentistry at ten o'clock, Monday, May 14.

The written theoretic examination Monday evening, May 14, and Tuesday, May 15.

Candidates holding temporary permits, and coming under the rules in force prior to January 1, 1900, must appear Monday, May 14, between the hours of ten A.M. and two P.M.

All persons desiring to practise dentistry in this State must apply to the Recorder for *revised rules* and for the proper blanks. Blanks must be carefully filled in and sworn to, and with the fee, twenty-five dollars, filed with the Recorder at least one week before the day of examination.

GEO. L. PARMELE, M.D., D.M.D.,
*Dental Commissioner and Recorder.*

HARTFORD, CONN., April 6, 1900.

---

### PENNSYLVANIA STATE DENTAL SOCIETY.

THE National Association having changed the date of its meeting, for this year, to July 10, the Pennsylvania State Dental Society will meet on July 5, 6, and 7, at Reading, Pa. By order of the Council.

ROBERT HUEY,
*President.*

---

### ILLINOIS STATE DENTAL SOCIETY.

AT the annual meeting of the Illinois State Dental Society, to be held in Springfield, Ill., May 8, 1900, the following papers will be read:

President's Address, Dr. R. N. Lawrence; Report of Committee on Dental Science and Literature, Dr. A. W. Harlan; Report of Committee on Dental Art and Invention, Dr. H. J. Goslee; Gold Crown with Solid Carved Cusps, Dr. J. E. Nyman; A few Thoughts on Prosthetic Dentistry, Dr. W. W. Moorhead; Simple Method of Treatment of Fractures of Lower Jaw, Dr. W. A. Johnson; Calcification a Controlling Factor in the Treatment of the Teeth, Dr. Grafton Monroe; Habits Incident to the Dental Profession, Dr. G. W. Ensminger; Electricity through the Ages and its Value with regard to Dentistry, Mr. G. E. Lob, M.E.; Pyorrhœa Alveolaris, So-called, Dr. A. H. Peck; Operative Dentistry: (1) To emphasize some things in Operative Procedures, Dr. D.

M. Cattell; (2) Subject to be announced, Dr. Edmund Noyes (it is expected that Dr. Black will open the discussion on both of these papers. The discussion on this subject not to be confined strictly to the papers read); Antiseptics, Dr. Elgin MaWhinny; Report of Supervisor of Clinics (the clinical work will be fully represented, twenty-five operators having signified their intention to be present), Dr. A. J. Hinkins.

---

### MISSOURI STATE DENTAL ASSOCIATION.

THE thirty-sixth annual meeting of the Missouri State Dental Association will be held July 10, 11, 12, and 13, 1900, at Louisiana, Mo. A cordial invitation is extended to all reputable dentists to attend.

B. L. THORPE,
*Corresponding Secretary.*

---

### CHICAGO DENTAL SOCIETY.

THE following list of officers of the Chicago Dental Society for 1900-1901 were elected at the annual meeting, held in the Stewart Building, Tuesday evening, April 3, 1900:

President, Geo. W. Cook; First Vice-President, Geo. B. Perry; Second Vice-President, H. J. Goslee; Secretary, Elgin Ma Whinney; Corresponding Secretary, C. S. Bigelow; Treasurer, A. B. Clark; Librarian, H. W. Sale; Member Board of Directors, J. E. Hinkins.

*Board of Censors.*—W. V.-B. Ames, Chairman, C. N. Johnson, A. W. Harlan.

C. S. BIGELOW,
*Corresponding Secretary.*

100 STATE STREET, CHICAGO, April 25, 1900.

